# Giardia lamblia infection: review of current diagnostic strategies

**Published:** 2019

**Authors:** Hossein Hooshyar, Parvin Rostamkhani, Mohsen Arbabi, Mahdi Delavari

**Affiliations:** *Department of Medical Parasitology, School of Medicine, Kashan University of Medical Sciences, Kashan, Iran *

**Keywords:** Giardia, Diagnosis, Methods, Test

## Abstract

Giardiasis has a global distribution and it is a common cause of diarrhea in both children and adults and is transmitted via the fecal-oral route through direct or indirect ingestion of cysts. The laboratory diagnosis of Giardia spp. is mainly based on demonstration of microscopic cyst or trophozoite in stool samples but several immunological-based assays and molecular methods are also available for giardiasis diagnosis. The aim of this study was to conduct a review of the applied methods in medical laboratory and to highlight pitfalls and challenges of them for diagnosis of giardiasis. In this article we have evaluated the Giardia diagnostic methods with a broad review of literature, electronic databases and books. The search has covered the articles and some textbooks that have published up to 2018.

It has been concluded that traditional microscopy combination with stool concentration method should still be held in the routine medical laboratory due to economical and high sensitivity and immunological-based assay and molecular methods which are recommended to use as a complementary test to the traditional technique.

## Introduction

 The etiological agent of Giardiasis, *Giardia* *duodenalis* (syn. *G. intestinalis, G. lamblia*) is one of the most prevalent intestinal protozoan flagellate of the human. The life cycle of *Giardia* species is simple and it is included of two active trophozoite and cystic forms.

This parasite transmits via fecal-oral route through direct or indirect ingestion of infectious cysts. The incubation period varies from 9 to 15 days after ingestion of cysts. Symptoms of infection are varied from the absence of symptoms to acute watery diarrhea, nausea, epigastric pain and weight loss (1,2). 

Giardiasis has a global distribution and it is common in both children and adults. The prevalence of *Giardia* infection is higher in developing countries. More than 200 million cases of giardiasis are annually diagnosed worldwide. Since 2004, *Giardia* has been included in the "neglected diseases initiative" by World Health Organization (3). The infection rate in asymptomatic children has been reported from 8% to 30% in developing countries and 1-8% in industrialized regions (4). The occurrence of giardiasis is probably higher in individuals with diarrhea.

The prevalence of human giardiasis in different regions of Iran has been reported from 1.2% to 38% (5). In immunocompromised patients, *Giardia* is not considered as an opportunistic pathogen causing prolonged symptoms and enteritis. In HIV-infected individuals, symptoms of giardiasis are similar to HIV-negative individuals and its prevalence has been reported between 1.5% and 17.7% (6). The prevalence of giardiasis was reported 3.1% in HIV/AIDS patients in Iran (7).

Correct diagnosis of giardiasis is important for treatment and prevention of diseases. The laboratory diagnosis of *Giardia* spp. is mainly based on finding and demonstration of microscopic cyst in stool samples, but immunological-based assay and molecular methods also are available and are used for diagnostic or research proposes in developed countries. All diagnostic methods provide different sensitivity and specificity. This condition depends on some factors such as the method of test, the skill of operations and the stage that the test has been performed (8). Since it is important for treatment and control of giardiasis which diagnosis method has been employed? Some methods that are accurate, cheap and relatively easy are required to routine laboratory diagnosis and for large-scale population screening. There are various studies that were carried out to introduce the suitable diagnostic method of giardiasis (8-10). The aim of this study was to conduct a review of the main methods which used in clinical and research laboratory for diagnosis of human giardiasis. 

## Methods

Manual and electronic searches in national and international electronic databases and journals have been performed to find the related data reporting on *Giardia* diagnostic methods. The search has covered the articles and some textbooks that have published up to 2018.

These articles had used at least one method such as stool examination, immunodiagnostic methods, Plymerase chain reaction (PCR) and culture for diagnosis of Giardiasis.

Electronic searching was performed in the international databases such as ISI Web of Science, PubMed, Scirus, EMBASE, Scopus, Science Direct and Google Scholar. The national databases searching were Iran Medex, Iran Doc, Magiran and Scientific Information Database. The following keywords: "*Giardia*", "diagnosis", "immunodiagnosis", "molecular" have been used as a panel of keywords. The search restricted to English and Persian languages and the references of selected papers were checked for more accuracy. 

## Results

**Diagnostic methods**

*Fecal microscopy examination*

The microscopic identification of *Giardia* spp. in fecal samples is considered as the gold standard method for the diagnosis of giardiasis. This method is performed to detecting cysts and trophozoites. The sensitivity of microscopy techniques depends on using direct or concentration methods, the number of examined fecal samples and employment of professionally trained persons (11,12).

*Direct examination methods*

The diagnosis of giardiasis in most cases is mainly confirmed by stool examination. Fecal suspension in physiological salt solution (0.85 NaCl) or fixation in sodium acetate–acetic acid–formalin (SAF) is used to prepare wet mounts in order to the observation of *Giardia* throphozoite in diarrhea or loose samples. Wet mounts smear can be examined either unstained or iodine stained (2-5% lugol’s solution).

Examination of direct wet saline preparation of a fresh stool specimen allows motile trophozoites to be seen, but in stained and SAF preparation smears the trophozoites will be non-motile. If diarrhea stool sample containing trophozoite left too long without fixations or preservatives solution, the organisms tend to degeneration, thus preventing has been recommended for sample transfer and protection of the typical trophozoite morphology. A number of commercial kits with preservative solutions are available or can be made manually. The most commonly used preservation kit contains of 10% buffered formalin, polyvinyl alcohol (PVA), merthiolate-iodine-formalin, and SAF solution (9, 13). Polyvinyl alcohol is suitable for preparation of smear in order to permanent staining.

In the asymptomatic individuals and healthy carrier who do not have diarrhea, the cyst stage is more likely to be seen in a fecal sample examination. Fecal suspension in saline or lugol’s solution or in a fixative solution may be used for cyst identification. 

**How many fecal examinations are necessary to detect Giardia cyst by wet mount methods?**

In these cases, the number of cysts may be low in the fecal specimens, thus the wet mount examination of stool samples may not detect the parasite. It has been recommended for preparation and examination of two or more, even to six wet mounts smear for increasing the chance of finding parasite agents. Also, it must be requested from patient to submit more than one stool samples on consecutive days due to the intermittent shedding of the cyst. Examination of one stool sample will allow the diagnosis of 60 to 80% of infections, two stool samples examination will allow the detection of 80 to 90%, and diagnosis will be over 90% if three stool samples have been examined (14). However, in some cases, the examination of more than three stool samples is necessary due to intermittent or low levels of cyst shedding (15). 

Direct microscopy method has been considered economical and rapid for the diagnosis in the medical diagnostic laboratory.

*Concentration methods*

 Fecal concentration is a recommended and routine procedure that allows the detection of a small number of *Giardia* cysts may be missed by using wet mounts direct smear. Concentration methods have been designed to separate protozoan cysts and helminthes eggs from excess fecal debris. Flotation and sedimentation are two types of concentration procedure that have been used in the parasitological laboratory. Flotation methods permit the separation of some protozoan cysts and helminthes eggs through the use of a liquid with high specific gravity such as NaCl, NaNO3, ZnSO4 (final specific gravity of about 1.20). Zinc sulfate has been recommended as the best saturated solution to detection of *Giardia* cyst (4). *Giardia* cysts and other parasitic elements are floated and visible on the surface and the debris aggregate at the bottom of the tube. A modified technique has been made by adding a centrifugation step after the samples emulsified in flotation methods for increasing the efficiency of cyst recovery. 

Yields of this technique are cleaner than sedimentation methods, but in flotation techniques the walls of cysts will often be collapsing.

The sedimentation procedures are the recommended methods as being the easiest to perform and less prone to technical errors (4). In this method, using centrifugation has been led to the recovery of *Giardia* cyst and other intestinal parasite in fecal sediment. These methods are the easiest but the preparation contains more debris. Many sedimentation methods have been employed for detection of *Giardia* spp. and another intestinal protozoan cyst. Among them, the formalin-ether/ formalin-ethyl acetate, sedimentation technique are best to employ and generally applicable. In these methods, 10% formalin has been fixed and preserved cyst stage and also provides user protection due to microbicidal activity of formalin. The ether or ethyl acetate has been used to remove the fat drop and oils. In this method, less distortion of *Giardia* cysts occurs in comparison with zinc sulfate flotation.

Comparison of wet mounts smear and formalin-ether concentration techniques in the diagnosis of intestinal parasite has showed that formalin-ether concentration technique detected 65.26% of positive specimens for one or more intestinal parasites while the direct wet mount smear was only 34.74% sensitivity (16). 

A significant number of the infected individual was missed by using wet mount smear method. Another study has showed 55% sensitivity for wet mount smear and 83% for formalin-ether concentration in *Giardia* cyst diagnosis at infected BALB/c mice (8). 

The formalin-ether concentration technique can be adopted and used as a routine method in medical diagnostic laboratories.

The Kato-Katz method is a sensitive method that has been widely used for diagnosis of Schistosoma mansoni ova and soil transmitted helminths such as, Ascaris, Trichuris, and Hookworms. 

Kato-katz is not used routinely for diagnosis of *Giardia* spp. and other human intestinal protozoa. However, this technique was evaluated by some researchers for detection of *Giardia* infection (17-18). Using this method for diagnosis of *Giardia* spp. has limitations, particularly in sensitivity.

Sucrose density gradient centrifugation is not normally and usually employed for *Giardia* spp. diagnosis in the medical laboratories. This method has been used for isolation of *Giardia* cyst from fecal debris. In this technique, debris was removed by centrifugation and re-suspension of infected stool specimen in 0.85 molars sucrose suspension (19). The high purified and viability of the cysts have allowed using this technique, for studies on cultural methods, excystation, and the effect of drug agents, molecular or biochemical characterization. This method is expensive, time-consuming and it is not economical that employing in medical diagnostic laboratories for cyst diagnosis. However, some studies have been used this method for diagnosis proposes and compare it with other techniques. Elmi *et al.* have reported a high sensitivity (94%) for this method compared to direct and formalin–ether methods. They suggested that sucrose density gradient is a suitable diagnostic method and it can be used in place of formalin-ether concentration (8).

Xiao and Herd showed that sensitivity of a sucrose gradient method is depended to intensities of sample infections, so they have reported 42.9% and 51.2% recovery rate of *Giardia* cyst for sucrose gradient flotation when infection intensities were moderate or high (20).

*Staining*

Identification of *Giardia* trophozoite and cyst are dependent on morphological criteria. Sometimes the correct identification of this morphological character may need to the examination of the permanent stained smear for revealing some details of the organism that cannot be seen in unstained or temporary stained smears. Several temporary and permanent stainings have been existed for identification and diagnosis of *Giardia* trophozoite and cyst. Although an experienced microscopic can identify this organism on a slide of concentrated and temporary stained sample, the permanent stain is not recommended for all stool samples that submitted for *Giardia* examination.

 A number of staining technique is available for staining of *Giardia* trophozoite or cyst. Temporary stain such as methylene blue and iodine or lugol solution are primarily stains that have been used after preparation of a wet mount or concentration smears for better detection.

Some permanent stains have been used for *Giardia* spp. diagnosis. Giemsa stain is an easy to use permanent stain for routine clinical laboratory use. In this staining, flagella and nuclei are reddish pink stain, and cytoplasm stains grey-blue. Iron hematoxylin is a useful staining procedure for demonstrating trophozoite and cyst of *Giardia*, additionally; automated staining machines can be used for this method (21-22). Although Chlorazol Black is not widely used, it is another stain that has been used for permanent staining of trophozoite and cyst of *Giardia* spp. and other intestinal protozoa. In this staining, the background of smear is light blue/grey, the cytoplasm of organism stains blur/grey and nuclei tend to dark (blue/black) (23-24).

Trichrome is a shorter permanent stain that is simple and well- stained smears in about 45 min to 1 hour. This procedure is of value for staining fresh faecal specimens as well as stool fixed in PVA. In this staining, the background materials stain green or blue-green. The cytoplasm of trophozoites and cyst stain green or greenish-blue, nuclei and nuclear chromatin stain red or red-purple (23).

*Culture methods*

Although cultivation of human intestinal protozoa is a useful method for detection and diagnostic purpose, routine culture techniques were not established for *Giardia* spp. in the clinical diagnostic laboratory. Cultivation of *Giardia* spp. is applied in the research laboratory for many types of studies that require a large number of trophozoite.

The *Giardia* spp. is grown in the monoxenic and xenic type of culture system. In monoxenic system, the parasite has been grown in the presence of a single additional flora organism species and in axenic, parasite has been grown in the absence of any other accompanied alive cell (25). Monoxenic cultivation is an introduction to xenic growth; however, *Giardia* spp. can be established directly into axenic media. 

 The most common and suitable used medium for *Giardia* axenic culture is Diamond's medium "TYI-S-33" which modified by Keister DB (25-26).

*String test (Entero-Test)*

In some cases of giardiasis that routine laboratory methods are unable to confirm infection, examination of fluids obtained from duodeno-jejunal by endoscopy or using string test (entro-test) may be useful for revealing the *Giardia* trophozoites (9,27). The Entero-Test consists of a lead-weighted gelatin capsule containing a length of nylon string 90 or 140 cm. After ingestion, the capsule dissolves and the nylon releases down into the duodenal area by peristaltic action. The string was left in this area for a recommended of 4 hours, the nylon string was withdrawn, the fluid from the bile-stained portion of the string was extracted and examined by direct microscopy or inoculated to the culture medium (27-28). Some studies have demonstrated that application of the string test resulted in an increase of the successful axenic cultivation of *Giardia* spp. than other detection methods (28-30).

Also, a drop of mucus can be fixed directly on the slide and used for permanent staining (9).

The value of Entero-Test to fecal examination for *Giardia* spp. diagnosis is little known and reported inconsistent. Some researchers have reported that Entero-Test is reliable and superior to stool examination for identification of *Giardia *spp. in human and dog (31-32) while others do not support it. Goka* et al.* showed that giardiasis was diagnosed in 73% of 229 patients with the first fecal specimen while it was found in only 44% of the patients via duodenal aspirates examination (9, 15). However, further studies need to investigate this difference.

*Immunodiagnostic tests*

A variety of antibody and antigen detection methods have been developed and used for immunodiagnostic of giardiasis during the last three decades. Nevertheless, immunodiagnostic of giardiasis is still has a complementary role for microscopy stool test in the diagnosis of giardiasis. Immunodiagnostic test for *Giardia* spp. diagnostic includes immunoassay techniques such as ELISA for antibody detection and methods dependent on detection of *Giardia* *intestinalis* antigens in human fecal specimens (33).

*Antibody detection*

Both cell-mediated and humeral immunorespons stimulated in human giardiasis (34- 35). The presence of IgM, IgG and secretary IgA humeral response to acute giardiasis has been noted previously (34, 36-37). In persons with acute giardiasis level of IgM antibody fall to levels of healthy persons between two or three weeks after drug treatment. This indicates that detection of IgM antibody may be a useful indicator for diagnosis of current infection. IgG antibody response may remain for up to 18 months after infection, so it has been applied in epidemiological studies (4). Smith *et al., *in 1984 showed specific IgG antibody response to trophozoite is detectable in 81% of infected asymptomatic *Giardia* and only in 12% of healthy control individuals (38).

It is well known that *Giardia* spp. induces a strong production of IgA antibody in human and animal infections. Secretary IgA (sIgA) as the predominant antibody has been detected in duodenal fluid and saliva samples of infected people. The production of secretary IgA has been developed during active giardiasis, so detection and monitoring this antibody may be a useful tool for serodiagnosis (39-40).

A study on *Giardia*-infected children in Egypt has showed that salivary and serum IgA and IgG responses against *G. duodenalis* infection were significantly higher than non-*Giardia* infected children (p<0.001) (40). 

A variety of assays such as Elisa, IFA, Western blot have been used for the serodiagnosis of giardiasis, but these methods may be problematic as the antibody may be detectable as long times after treatment of acute diseases. Commercially produced kits were not developed for detection of serum antibodies to *Giardia* infection.

*Antigen detection*

Some methods include immunoassay techniques (including enzyme-linked immunosorbent assays [ELISAs], and rapid antigen detection tests [RDTs] such as non-enzymatic immunochromatographic assays) have been used to detect fecal antigens in both preserved formalin- and fresh stool specimens (33).

Several commercially kits are available and ELISAs are the basis for detection of faecal *G. intestinalis* antigens. Using immunoassay kits have been described for simultaneous detection of *Giardia* and *Cryptosporidium* or *Giardia* spp., *Cryptosporidium* and *Entamoeba* species antigens in faecal specimens (33, 41, 10). There are many published articles about comparing the sensitivity of immunoassays methods and the faecal microscopy in diagnosing *Giardia* infection. The overall conclusion of them with some exceptions is that immunoassay is more sensitive than or as sensitive as, microscopy fecal examination (33, 10, 42- 43).

**Table 1:** Comparison of sensitivity and specificity stool antigen detection kits for *Giardia* diagnosis

Authors	Year	Kit	Assay	Sensitivity%	Specificity%	References
Aldeen et al.	1998	Alexon pro spect microplate *Giardia*	Elisa	100	100	
Aldeen et al.	1998	Alexon pro spect new	Elisa	100	100	
Aldeen et al.	1998	Pro Spect T *Giardia* Rapid	Elisa	90	100	
Aldeen et al.	1998	Pro Spect T Giardia EZ microplate	Elisa	95.7	100	
Aldeen et al.	1998	Cambrigde microwell	Elisa	88.6	100	55
Aldeen et al.	1998	Meridian Premier	Elisa	92.9	100	
Aldeen et al.	1998	Trend G.lamblia direct detection system	Elisa	98.6	99.3	
Aldeen et al.	1998	Trend G.lamblia direction RS system	Elisa	97.1	100	
Garcia LS et al.	2000	Bio Site diagnosis	EIA	95.9	97.4	
Faubert G	2000	Manual-Non commerical	Elisa	79	_	
Faubert G	2000	Manual-Non commerical	CIE	90	_	
Schunk M et al.	2001	R-Biopharm RidaScreen® *Giardia*	EIA	100	99.6	
Duque-Beltrán, et al.	2002	Manual-Non commerical	Elisa	100	95	
Garcia LS, et al	2003	Immuno Card STAT	EIA	93.5	100	
Weitzel T, et al	2006	Ridascreen *Giardia*	EIA	82	99.4	
Weitzel T, et al	2006	Rida Quick *Giardia*	EIA	80	100	
Weitzel T, et al	2006	Rida Quick Combi	EIA	80	98.9	
Weitzel T, et al	2006	*Giardia*-Strip	ICT	44	100	
Mekaru SR, et al.	2007	SNAP Giardia	EIA	85.3	100	
Mekaru SR, et al.	2007	Pro Spect T *Giardia *microplate assay	EIA	91.2	99.4	
Mekaru SR, et al.	2007	Immuno Card Stat	EIA	72.7	99	
Mekaru SR, et al.	2007	X pect	EIA	79.4	99	
Schuurman T, et al.	2007	Immuno Card Stat Rapid assay	Elisa	98	97	
Al-Saeed, Issa SH.	2010	Strip, Novum Diagnostica	Elisa	76.4	100	
Goni P, et al.	2012	R-Biopharm RidaScreen® *Giardia*	ICT	90-97	99	
Minak J, et al.	2012	Immuno Card STAT	EIA	78	44	
Minak J, et al.	2012	Xpect	EIA	56	78	
Bouyou-Akotet et al.	2016	Immuno Card STAT	EIA	63	96.6	
Beyhan YE, Taş Cengiz Z	2017	*Giardia* CELISA	Elisa	53.3	79	
Goudal A, et al.	2018	*Crypto/Giardia* K-SeT®, Coris ioconcept	ICT	86.7	100	
Uiterwijk M, et al.	2018	IDEXX SNAP *Giardia*®	EIA	71.9	99.6	
Barbecho J M, et al	2018	ProSpecT Microtiter Plate	ELISA	94.1	97.4	
Barbecho J M, et al	2018	SNAP *Giardia*	ELISA	87.1	93.4	
Barbecho J M , et al	2018	Anigen Rapid CPV-CCV-*Giardia *Antigen Test	ELISA	80.2	80.3	
Barbecho J M, et al	2018	Witness *Giardia *Test	ELISA	73.3	71.1	
Barbecho J M, et al	2018	VetScan Canine *Giardia* Rapid Test	ELISA	70	85.5	
Hijjawi N, et al	2018	RidaScreen® *Giardia*	ELISA	76.5	68	

EIA: Enzyme immunochromatographic assay; ELISA: enzyme-linked immunosorbent assay CIE: Counter immuno electrophoresis; ICT:Immuno chromatographic Test

**Table 2:** Comparison of sensitivity and Specificity of different method in *Giardia *diagnosis

**Methods**	**Sensitivity %**	**Specificity %**	**References**
Direct stool examination	34.7-55	96-100	8, 16, 68
Stool concentration	65.2-83	85-97	8, 16, 60, 67
Sucrose density gradient	42-94	97-100	8, 20
String test (Entero-Test)	44-73	97-100	9, 15, 32
Antigen detection	44-100	68-100	34, 46, 55-67
Molecular assay	58-92	56-100	51-54

One of the best antigens that have ever been used is *Giardia* stool antigen with a relative molecular mass of 65 Kda (GSA65) which presenst in both trophozoites and cysts (4). The reported sensitivity of Elisa-GSA65 for a single specimen varies between 95 and 100% with 100% specificity. Elisa-GSA65 can detect giardiasis in at least 30% more cases than microscopy examination (44).

There are some non-enzymatic immunochromatographic techniques for identification of *G. intestinalis* antigen in faecal specimens. In these methods the captured antigen is detectable with an antibody conjugated to a visible marker. The presence of *G. intestinalis* antigen indicated by a dark band and it is visible to the naked eye (4, 33.^11^-^12^, ^45^- ^46^). 

Results of immunochromatographic techniques are visible in 10–15 min, in contrast to the longer time that required enzyme-linked immunosorbent assays.

Considering the cost of antigen detection tests, feacal microscopic which has been used in medical laboratories examination is cheaper and easier.

The sensitivity and specificity of different kits for Giardia stool antigen detection were compared in Table1.

*Molecular methods*

Molecular diagnosis of giardiasis is not used in routine medical laboratories.

PCR-based methods are often restricted to research laboratories and mostly used for sub-typing propose such as determination of assemblages or sub-assemblages of Giardia duodenalis (4, 5). The major 

target gene sequence which has been used in different molecular studies of Giardia species are genes encoding small subunit (SSU) ribosomal RNA, glutamate dehydrogenase (gdh), triosephosphate isomerase (tpi) and β-giardin genes (a protein in the adhesive disk of *Giardia*).

comparison and polymor-phisms of glutamate dehydrogenase (gdh), the small-subunit of ribosomal RNA (SSU), and triosephosphate isomerase (tpi) genes, showed that *G. duodenalis* is classified to at least eight distinct genetic groups (A to H) or assemblages (1, 5). All these assemblages are indistinguishable by light microscopy. Two assemblages A and B are mainly isolated from human. Genotyping study of human isolates of *Giardia* in different regions of Iran and neighboring countries indicated that AII as the most common sub-assemblage is followed by BIII and BIV, respectively (5, 19).

Using multiplex real-time PCR have been described for the simultaneous detection of *Giardia* spp., *Cryptosporidium,* *Dientamoeba* and *Entamoeba histolytica *with a high sensitivity and specificity (47). In recent years PCR-based methods have been used for detecting *G. intestinalis* and other human parasites in environmental sources such as water, and sewage (48-50).

There is an extensive literature that compares the molecular methods and other diagnostic technique in diagnosing *Giardia* infection (33,47-52). Real-time PCR has been reported to be more sensitive and beneficial than Elisa and faecal microscopy for diagnosing *G. intestinalis* infection (51). Using a real-time PCR-based as routine parasitological examination for the identification of *G. intestinalis* displayed an average 92%sensitivity and 100% specificity (53). Comparison of five diagnostic tests for identification of *Giardia duodenalis* in dog fecal samples has showed that performance of the PCR was poor and the relative sensitivity was 58% and specificity reported 56% (54). 

A recent study by Hijjawi *et al.* (2018), the sensitivity and specificity for the Nested-PCR in diagnosis of giardiasis was 89.9% and 82.9% respectively (52).

## Conclusion

*Giardia* spp. is one of the most common waterborne parasites that infected human. Cyst stage of this parasite has been identified in surface waters such as rivers, lacks, and ponds. Contaminated food and water to *Giardia* cysts by the food-handlers can be one of the most important sources of transmission of this parasite to humans (5). The main symptoms of human acute giardiasis are diarrhea, flatulence, epigastric cramps, nausea, vomiting and weight loss (6).

It is well known that no traditional or new methods can detect all cases of *Giardia* infection. Several immunodiagnostic tests of rapid diagnosis of giardiasis have been developed particularly in the last three decades, mainly based on the detection of *Giardia* antigens in faecal specimens. While to the high sensitivity of these methods (Table 2), microscopy stool examination especially using concentration methods, most frequently has performed laboratory procedure worldwide as a good performance diagnostic strategy and should still be held as the golden standard. Non-morphological diagnostic methods particularly immunoassay is recommended to detect coproantigen is recommended as a complementary test to the traditional technique and has been applied in larger laboratories that process a large number of stool samples daily. The stool concentration techniques such formalin-ether method can be used as a routine and economical method in medical diagnostic laboratories in developing countries.
